# Knowledge and perceptions of the Sustainable Development Goals (SDGs) among medical students at King Saud University: a cross-sectional study

**DOI:** 10.3389/fpubh.2026.1745983

**Published:** 2026-03-11

**Authors:** Nurah Alamro, Abdullah AlDhuwaihy, Talal Alghadir, Faisal Alshuwaier, Mohammad Alhudaithi, Yazeed B. AlSulaim, Abdullah Saud AlOsaimi

**Affiliations:** 1 Department of Family and Community Medicine, College of Medicine, King Saud University, Riyadh, Saudi Arabia; 2 Department of Family and Community Medicine, King Saud University Medical City, Riyadh, Saudi Arabia; 3 College of Medicine, King Saud University, Riyadh, Saudi Arabia

**Keywords:** cross-sectional study, knowledge, medical education, perception, Saudi Arabia, SDGs

## Abstract

**Background:**

The Sustainable Development Goals (SDGs) represent a global agenda to promote social, economic, and environmental development by 2030. Medical students, as future healthcare providers, have a critical role in advancing these goals, particularly Goal 3: Good Health and Wellbeing. This study assessed knowledge and perceptions of SDGs among medical students at King Saud University in the context of Saudi Arabia's Vision 2030.

**Methods:**

A cross-sectional study was conducted between December 2024 and April 2025, targeting undergraduate medical students across all academic years. Stratified random sampling was employed, and data were collected using a validated online questionnaire that assessed demographics, knowledge, and perception. Statistical analysis included descriptive statistics, Chi-square tests, and logistic regression [SPSS version 29.0 (IBM Corp., Armonk, NY, USA)], with significance set at *p* ≤ 0.05.

**Results:**

A total of 425 students participated (55.5% male; median age 21). Good knowledge of the SDGs was demonstrated by 54.6% of participants, while 64.9% showed positive perceptions. Knowledge levels improved with advancing academic year. Female students showed significantly more favorable perceptions of the SDGs. While younger students ( ≤ 21 years) initially appeared more favorable in unadjusted analysis, this association was not independently significant after adjustment.

**Conclusions:**

While overall awareness of the SDGs among medical students was acceptable, gaps in detailed knowledge remain. Early and structured integration of SDG-related content into the medical curriculum is recommended to foster deeper understanding and engagement, thereby preparing future physicians to contribute more effectively to sustainable development efforts.

## Background

In 2015, the United Nations adopted the 2030 Agenda for Sustainable Development. The agenda included 17 sustainable development goals and 169 targets and sought to improve upon the earlier millennium development goals. The SDGs address a wide range of social, economic, and environmental issues, including poverty, health, education, gender equality, and sustainability. Achieving the SDGs requires the engagement of students and future professionals, making their awareness and understanding of the goals essential. Medical students, as future healthcare providers, will be key stakeholders in SDG implementation, making it all the more important to ensure they are knowledgeable and have an accurate perception of SDGs (1). The SDGs are aligned with Saudi Arabia's Vision 2030, which promotes sustainable development through initiatives such as health system reform (SDG 3), educational modernization (SDG 4), and economic diversification (SDG 8) (2). SDG 3, “Good Health and Wellbeing,” focuses on reducing maternal and child mortality, combating communicable and non-communicable diseases, and strengthening health systems. These priorities are directly relevant to medical education and the future role of healthcare professionals. Thus, SDGs are of great importance to medical professionals as highlighted in the agenda for sustainable development (1). This underscores the importance of integrating SDG content into medical education curricula (3). Evidence from Saudi Arabia and the Gulf region assessing SDG-related knowledge and perceptions among undergraduate medical students remains limited, supporting the need for the present study. To date, there is no clear evidence describing the level of SDG familiarity among undergraduate medical students at King Saud University. Therefore, the extent of students' awareness and perceptions remains uncertain, which represents an important local knowledge gap and provides a clear justification for conducting the present study. SDG knowledge is particularly important for medical students because physicians influence health outcomes beyond clinical encounters through prevention, public health advocacy, and leadership within health systems. Several SDGs are directly connected to health determinants (e.g., poverty, education, clean water, and sustainable communities) and to health-system goals such as equity and universal access. Therefore, introducing SDG concepts during undergraduate medical education can help prepare future physicians to recognize and address the broader determinants of health in their practice and community roles. This study is particularly relevant in Saudi Arabia because Vision 2030 prioritizes health-sector transformation, prevention, improved quality of life, and sustainable national development—priorities that closely align with the SDG framework. As future physicians, undergraduate medical students will contribute to these priorities through preventive care, public health advocacy, and health-system improvement initiatives. Therefore, assessing SDG-related knowledge and perceptions among medical students provides timely evidence to inform educational strategies that support Vision 2030–aligned health workforce development. If medical students lack awareness of the SDGs, this may limit their ability to integrate a broader public health and prevention perspective into future practice, including recognition of social and environmental determinants of health. Limited SDG literacy may also reduce readiness to contribute to health equity initiatives, community engagement, and health-system improvement programs aligned with national priorities. Therefore, assessing SDG knowledge and perceptions during undergraduate training is important to inform educational interventions that prepare future physicians for these expanded professional responsibilities. This study primarily aims to estimate the levels of knowledge and perceptions toward the SDGs among medical students at the College of Medicine, King Saud University, during the 2024–2025 academic year. Additionally, it seeks to examine the association between selected demographic factors, such as age, gender, and students' perceptions of the SDGs. A further objective is to explore the relationship between students' academic year and their level of knowledge about the SDGs.

## Methods

### Study design

This cross-sectional study was conducted at the College of Medicine, King Saud University, in Riyadh, Saudi Arabia, between December 2024 and April 2025.

### Inclusion and exclusion criteria

All medical students currently enrolled at the College of Medicine, King Saud University, aged 18 years or older, of either gender, and spanning the first through fifth academic years, were eligible for participation.

### Study variables

Independent variables included age, gender, academic year, and GPA. Dependent variables were students' knowledge and perception levels regarding the SDGs.

### Sample size estimation

The sample size was determined using the standard formula for estimating a population proportion. Based on an anticipated knowledge prevalence of 70% from previous studies in Malaysia and Nigeria (4–6), a 95% confidence interval, and a 5% margin of error, the minimum sample size calculated was 323 students. In order to compensate for an expected non-response rate of 20%, the final target was modified to be 404 students. Stratified random sampling was used to select the target sample size randomly from first-, second-, third-, fourth-, and fifth-year medical students. From each academic year, 82 students were randomly selected, 41 males and 41 females, using stratified sampling based on the attendance list. This stratified sampling was obtained from a total population of approximately 1,450 undergraduate medical students enrolled across all five academic years at King Saud University during the study period. Ten sets of 41 random numbers were generated from the serial numbers in each year's attendance list. Using these random numbers, the study subjects were identified through their university ID numbers.

### Data collection

Recruitment was carried out by distributing a self-administered online questionnaire to the selected participants via the university's official student communication channels, including email and SMS to their registered mobile numbers. The data collection tool was a structured Google Forms survey consisting of 15 questions divided into three sections: Demographics, Knowledge, and Perception. In this study, perception was defined as medical students' cognitive views regarding SDG achievability and comparative appraisal (SDGs vs. MDGs), assessed using two 5-point Likert-scale items. We used a previously validated questionnaire (4), which has been published elsewhere. Minor contextual adaptations were made for Saudi medical students, while retaining the structure and validity of the original instrument. Some questions of the questionnaire were adapted to suit our study population. The survey took approximately 2 min to complete. Data were collected anonymously without personal identifiers to ensure participant confidentiality. Participants provided electronic informed consent at the beginning of the survey. The questionnaire required students to respond to all items before submission. Thus, no responses had missing values, and no data imputation was required. All responses were stored securely, kept under password protection and only accessed by the study authors.

### Data analysis

Knowledge score (0–7) was dichotomized as good knowledge (≥3 out of 7) based on benchmarks from previous published studies in Malaysia and Nigeria (4–6), and poor knowledge (< 3). Perception was calculated as the mean score of the two 5-point Likert-scale items (1 = strongly disagree to 5 = strongly agree): (1) “I believe the SDGs will be achieved” and (2) “I think the SDGs are better than the Millennium Development Goals (MDGs).” The overall perception score for each student was computed as the mean of the responses to these two items. Students were classified as having positive perception if their mean score was equal to or greater than the median of the overall perception scores in the sample (median = 3.0). Age was dichotomized as ≤ 21 years vs. ≥22 years based on the sample median to create balanced groups for regression analysis. Descriptive statistics were used for all variables. Associations between categorical variables were assessed with Chi-square tests and odds ratios. Logistic regression was used to identify predictors of knowledge and perception. Univariate (bivariate) analyses were first conducted to identify variables potentially associated with knowledge and perception outcomes. Variables demonstrating associations at a threshold of *p* < 0.25 in the univariate analyses were subsequently entered into multivariable logistic regression models to identify independent predictors while controlling for potential confounders. Bivariate (crude) logistic regression was employed first. Multivariable logistic regression was then performed, adjusting for age, GPA, and academic year to identify independent predictors of knowledge and perception. For continuous variables, Pearson correlation and linear regression were applied. A *p*-value ≤ 0.05 with 95% CIs was considered statistically significant. Analyses were conducted in SPSS version 29.0 (IBM Corp., Armonk, NY, USA).

## Results

### Participant characteristics

A total of 425 medical students from the College of Medicine at King Saud University participated in the study. The sample included 55.5% male and 44.5% female students.

The median age of the participants was 21 years (IQR = 3.0 years). The academic year distribution was as follows: first-year students comprised 16.7% of the sample, second-year students 17.9%, third-year students 20.2%, fourth-year students 21.6%, and Fifth-year students formed the largest group (23.5%). Regarding academic performance, the majority of students (64.7%) had a GPA between 4.5 and 5.00, while 26.1% had a GPA ranging from 4.00 to 4.49. Only 9.2% of students reported a GPA below 4.00 (Table 1).

**Table 1:** Sociodemographic characteristics of medical students at KSU (*N* = 425).

**Item**	***n* (%)**
**Gender**	
Male	236 (55.5)
Female	189 (44.5)
**Age in years, median (IQR** ^**a**^ **)**	21 (3.0)
**Academic year**	
First-year	71 (16.7)
Second-year	76 (17.9)
Third-year	86 (20.2)
Fourth-year	92 (21.6)
Fifth-year	100 (23.5)
**GPA** ^b^	
4.5–5.00	275 (64.7)
4.00–4.49	111 (26.1)
Below 4.00	39 (9.2)

^a^IQR interquartile range. ^b^GPA grade point average.

### Knowledge and perception scores

The mean knowledge score (0–7) was 3.67 (SD = 0.70), with 54.6% of students displaying good knowledge of the SDGs, good knowledge was defined as a score ≥3, consistent with previous studies (4–6). For perception, the median overall score on a 5-point Likert scale was 3.0 (IQR = 1.0), with 64.9% of students displaying positive perception about the SDGs. Regarding key perception statements, Students' opinions varied across perception items. For example, 22.4% of students strongly agreed, and 38.1% agreed with the statement “I believe the SDGs will be achieved.” Meanwhile, 35.8% expressed a neutral stance, 2.8% disagreed, and 0.9% strongly disagreed. Similarly, in response to the statement “I think the SDGs are better than the Millennium Development Goals (MDGs),” 15.1% strongly agreed, 27.5% agreed, 56.2% expressed a neutral stance, 0.9% disagreed, and 0.2% strongly disagreed (Table 2).

**Table 2:** Knowledge and perception scores of medical students (*N* = 425).

**A. Overall scores**		Mean ± SD^**a**^		Median (IQR^**b**^)	
**Knowledge score (0–7)**		3.67 (0.70)		–	
**Overall perception score (1–5 Likert mean)**		–		3.0 (1.0)	
**B. Distribution of responses to key perception statements**	**Strongly agree (%)**	**Agree (%)**	**Neutral (%)**	**Disagree (%)**	**Strongly disagree (%)**
**Perception statement**					
I believe the SDGs will be achieved.	95 (22.4)	162 (38.1)	152 (35.8)	12 (2.8)	4 (0.9)
I think the SDGs are better than the MDGs.	64 (15.1)	117 (27.5)	239 (56.2)	4 (0.9)	1 (0.2)

^a^SD standard deviation. ^b^IQR interquartile range.

### Comparison of knowledge and perception across groups

Bivariate regression analysis revealed no significant association between gender and knowledge levels (*p* = 0.973). However, gender was significantly associated with perception; using males as the reference group, females had significantly higher odds of positive perception (OR = 2.300, 95% CI: 1.513–3.495, *p* < 0.001).

Age did not have a statistically significant association with knowledge levels (*p* = 0.288). Although students aged 21 years or younger initially demonstrated higher positive perceptions in unadjusted analysis, this association did not remain significant after adjustment for gender, GPA, and academic year. This suggests that age may have acted as a confounder.

Academic year had a strong association with both knowledge and perception. Compared to second-year students (reference group), third-year students had 2.45 times higher odds of having good knowledge (OR = 2.450, 95% CI: 1.301–4.612, *p* = 0.006), while fourth-year (OR = 1.887, 95% CI: 1.019–3.493, *p* = 0.043) and fifth-year students (OR = 2.177, 95% CI: 1.186–3.998, *p* = 0.012) also showed significantly higher knowledge levels. Perception was significantly more positive among first-year students (OR = 3.190, 95% CI: 1.643–6.191, *p* = 0.001), second-year students (OR = 2.659, 95% CI: 1.413–5.004, *p* = 0.002), third-year students (OR = 2.367, 95% CI: 1.298–4.318, *p* = 0.005), and fourth-year students (OR = 2.239, 95% CI: 1.246–4.025, *p* = 0.007) compared to fifth-year students, who served as the reference group (Table 3).

**Table 3:** Comparison of knowledge and perception across groups (Bivariate Regression).

			Knowledge				Perception	
**Group**	**Good knowledge (%)**	**Poor knowledge** ***n*** **(%)**	**Odds ratio (CI 95%)**	***P*** **-value**	**Positive perception** ***n*** **(%)**	**Negative perception** ***n*** **(%)**	**Odds ratio (CI 95%)**	***P*** **-value**
**Gender**								
Male	129 (54.7)	107 (45.3)	1.0 (ref.)	-	134 (56.8)	102 (43.2)	1.0 (ref.)	-
Female	103 (54.5)	86 (45.5)	0.993 (0.676–1.459)	0.973	142 (75.1)	47 (24.9)	2.300 (1.513–3.495)	< 0.001
**Age**								
≤ 21	113 (52.1)	104 (47.9)	0.813 (0.554–1.192)	0.288	154 (71.0)	63 (29.0)	1.723 (1.152–2.577)	0.008
≥22	119 (57.2)	89 (42.8)	1.0 (ref.)	-	122 (58.7)	86 (41.3)	1.0 (ref.)	-
**Academic year**								
First- year	35 (49.3)	36 (50.7)	1.411 (0.735–2.710)	0.301	53 (74.6)	18 (25.4)	3.190 (1.643–6.191)	0.001
Second-year	31 (40.8)	45 (59.2)	1.0 (ref.)	-	54 (71.1)	22 (28.9)	2.659 (1.413–5.004)	0.002
Third- year	54 (62.8)	32 (37.2)	2.450 (1.301–4.612)	0.006	59 (68.8)	27 (31.4)	2.367 (1.298–4.318)	0.005
Fourth-year	52 (56.5)	40 (43.5)	1.887 (1.019–3.493)	0.043	62 (67.4)	30 (32.6)	2.239 (1.246–4.025)	0.007
Fifth- year	60 (60.0)	40 (40.0)	2.177 (1.186–3.998)	0.012	48 (48.0)	52 (52.0)	1.0 (ref.)	-
**GPA**								
4.50–5.00	145 (52.7)	130 (47.3)	1.0 (ref.)	–	183 (66.5)	92 (33.5)	1.243 (0.622–2.484)	0.538
4.00–4.49	66 (59.5)	45 (40.5)	1.315 (0.841–2.056)	0.230	69 (62.2)	42 (37.8)	1.027 (0.485–2.175)	0.945
Below 4.00	21 (53.8)	18 (46.2)	1.046 (0.534–2.049)	0.896	24 (61.5)	15 (38.5)	1.0 (ref.)	-

OR, odds ratio; CI, confidence interval; GPA, grade point average. Reference categories are indicated in the table as “ref.”

After adjusting for confounders, academic year remained an independent predictor of knowledge after adjusting for age, gender, and GPA. Students in third (AOR = 2.719, 95% CI: 1.434–5.433, *p* = 0.003), fourth (AOR = 3.567, 1.148–11.085, *p* = 0.028), and fifth-year (AOR = 4.312, 1.368–13.586, *p* = 0.013) demonstrated higher knowledge levels compared to second-year students, who served as the reference group, independently significant of age, gender, and GPA.

For perception, first-year students showed the highest positive perception (AOR = 4.335, 95% CI: 1.213–15.486, *p* = 0.024). Third-year students (AOR = 2.942, 95% CI: 1.005–8.612 *p* = 0.049) and fourth-year students (AOR = 1.861, 95% CI: 1.011–3.413 *p* = 0.046) also showed higher positive perception, independently significant to age, gender, and GPA, compared to fifth-year students, who served as the reference group. Female gender remained significantly associated with higher positive perception (AOR = 2.195, 95% CI: 1.412–3.414, *p* < 0.001) independently of age, academic year, and GPA. Notably, age was not significantly associated with perception after adjusting for gender, GPA, and academic year, suggesting it may have acted as a confounder in the unadjusted analysis (Table 4).

**Table 4:** Comparison of knowledge and perception across groups (Multivariate Regression).

	Knowledge			Perception		
**Group**	**Adjusted odds ratio**	**95% confidence interval**	***P*** **-value**	**Adjusted odds ratio**	**95% confidence interval**	***P*** **-value**
**Gender**						
Male	1.0 (ref.)	–	–	1.0 (ref.)	–	–
Female	0.919	0.611–1.382	0.685	2.195	1.412–3.414	< 0.001
**Academic year**						
First-year	1.474	0.752–2.887	0.258	4.335	1.213–15.486	0.024
Second-year	1.0 (ref.)	–	–	2.992	0.878–10.197	0.080
Third-year	2.719	1.434–5.433	0.003	2.942	1.005–8.612	0.049
Fourth-year	3.567	1.148–11.085	0.028	1.861	1.011–3.413	0.046
Fifth-year	4.312	1.368–13.586	0.013	1.0 (ref.)	–	–

Reference categories are indicated in the table as “ref.”

## Discussion

This study aimed to assess the knowledge and perception of SDGs among medical students at King Saud University. The findings show a moderate level of knowledge (mean score 3.67 out of 7). Only 54.6% of students possessed a good level of knowledge, which is lower than the 70% reported in earlier studies (4–6). Students also displayed a varied range of perceptions, with 60.5% students expressing agreement regarding the achievability of the SDGs compared to our hypothesized figure of 75%. These results indicate lower knowledge and less positive perceptions compared to earlier studies in Malaysia and Nigeria (4–6). In those settings, awareness was relatively high, but detailed knowledge of goals and targets remained limited. This lack of in-depth knowledge regarding the SDGs has been observed in our study population, and may contribute to the neutrality of students' perceptions highlighted in the question comparing the SDGs to the Millenium Development Goals. Neutral responses may reflect gaps in detailed knowledge, as also reported in other studies (4, 7, 8). We used the term “perception” because our items specifically captured students' views regarding SDG achievability and the comparative appraisal of SDGs vs. MDGs, rather than a broader attitudinal construct. While many KAP studies describe this domain as “attitude” [e.g., (5, 6)], our measurement focused on these two perception-oriented statements. Future work could incorporate a more comprehensive attitude domain to improve comparability with KAP-based frameworks.

A key finding was the relationship between academic year and knowledge level. The proportion of Senior students (third, fourth, and fifth years) possessing a high level of knowledge was 33.0% higher than their junior counterparts, consistent with findings from Indonesia and Nigeria, where academic exposure was shown to influence SDG awareness and comprehension (6, 9, 10). Although this figure is below 50%, it still likely reflects the effect of increased academic exposure and public health content.

Notably, perception levels were higher among first-year students compared to their seniors. This may indicate that junior medical students hold a more idealistic view (11); however, future qualitative research is warranted to confirm the underlying reasons. This phenomenon was also noted in a study in Ghana (11), where younger or newer students expressed stronger interest in SDG-related education. This finding warrants further investigation to identify the reasons for the decline in perception levels with advancing academic year.

We also found no significant association between gender and knowledge level (*p*-value = 0.973), Contrary to some literature suggesting female advantage, our study found no significant gender association with knowledge. Our results do not reflect the small, albeit statistically significant association between female gender and a higher level of knowledge about SDGs found in the literature (6, 9). Further investigation is warranted to determine the cause of this discrepancy.

Female students in our study were found to have significantly more positive perceptions toward SDGs than male students, aligning with findings from Indonesia and other parts of Nigeria that noted similar gender-based differences in SDG attitudes (6, 9).

Our findings add to the growing evidence in the literature supporting the integration of SDG content into medical curricula. Several studies, including those by McCormack et al. (3) and WHO initiatives, emphasize that health professions' education should reflect global health challenges and equip students with a framework for understanding sustainability in healthcare (12, 13). Programs such as those described in Mexico and by UNESCO's Global Action Program demonstrate successful models for embedding SDGs in higher education through interdisciplinary and participatory learning strategies (14, 15).

However, the study also highlights several gaps. Although general awareness is acceptable, in-depth understanding remains suboptimal, a limitation echoed in multiple regional and international studies (4, 7, 8). These knowledge gaps can hinder future physicians' ability to engage effectively with global health goals. Despite the importance of including the SDGs in medical curricula, several obstacles have been identified in the literature. Both (13) and (16) reported curriculum overcrowding as a major barrier to embedding sustainability teaching in medical curricula. Another obstacle, identified by Tun (13), was the absence of standardized evaluation metrics, which poses a challenge to assessing SDG-related learning outcomes. The moderate knowledge levels observed in our study, despite generally positive perceptions, may therefore be partly explained by these curricular and assessment challenges, underscoring the need for structured and measurable integration of SDG education within medical curricula.

### Strengths and limitations

This study's cross-sectional design limits the ability to infer causation or track changes over time. The use of self-reported data may introduce bias, and the single-institution setting may affect the generalizability of the findings. In addition, responses may have been influenced by social desirability bias, with students potentially overstating their knowledge or positive perceptions. The questionnaire included only 15 items, which may limit the depth of construct measurement compared to more comprehensive tools. Furthermore, while the instrument used was adapted from validated tools, its sensitivity to local and medical contexts could be improved in future research, for example by including items tailored to local medical education or adding scenario-based knowledge questions. Moreover, the limited number of questions pertaining to perception leaves room for a more thorough, multifaceted evaluation of perception. As the questionnaire was not designed as a multi-item psychometric scale, internal consistency metrics such as Cronbach's alpha were not calculated, which should be considered when interpreting the findings. Additionally, the high response rate and use of stratified random sampling enhance representativeness. Further multicenter studies in Saudi Arabia and the Gulf region are needed to assess SDG-related knowledge and perceptions among undergraduate medical students and to inform curriculum development.

### Recommendations

Firstly, further research using a cohort study design is warranted to follow changes in knowledge and perception of SDGs across the same group as they progress through their medical education. Such a study would also help establish a causal link between the variables. Secondly, the development of a more thorough and extensive validated tool for the evaluation of student knowledge and perception would allow for more precise characterization of areas of strength and weakness amongst students. Furthermore, the addition of a section to evaluate students' attitudes toward the SDGs would enhance educators' knowledge about the possible barriers that may be faced when educating students about the SDGs.

### Implications

Our findings underline the need for early, structured, and context-specific SDG education within the medical curriculum. Targeted efforts are needed to enhance not only awareness but also deep understanding of SDG targets and their practical applications in clinical and community settings. Interdisciplinary learning, case-based approaches, and partnerships with global health organizations could strengthen student engagement with sustainability principles (16–18). In a mixed-methods study among medical students in Sweden (19). reported that many medical students perceived their education on the SDGs/Agenda 2030 as limited, with SDG content often described as superficial or not explicitly linked to the SDG framework. Senior medical students described variable exposure, where SDGs were sometimes only briefly mentioned in selected lectures, while more in-depth learning was more likely through elective global health–related courses. Importantly, medical students also highlighted a need for clearer connections between the SDGs and day-to-day medical practice, noting that the relevance to the physician's role was not always made explicit. These findings support our recommendation that SDG content should be integrated early and taught explicitly within the medical curriculum using clinically relevant examples. These findings align with Saudi Arabia's Vision 2030, which emphasizes the importance of sustainable development and human capital investment, particularly through integrating global health priorities like the SDGs into educational and healthcare reforms.

## Conclusion

This study highlights that medical students at King Saud University demonstrated moderate knowledge and generally positive perceptions of the SDGs. Female and first-year students showed the most positive perceptions of the SDGs, while knowledge scores increased progressively from first-year to fifth-year students. This reflects a gap between surface-level awareness and in-depth knowledge, which may limit students' ability to engage meaningfully with global health and sustainability issues in their future practice.

The strong association between academic year and both knowledge and perception suggests the need for early and consistent integration of SDG-related content into the medical curriculum. Such integration should begin in the first academic year and include both didactic and applied components. This approach would enhance students' understanding of global health priorities. It would also foster a sense of responsibility toward sustainable development. Educational strategies such as case-based learning, interdisciplinary collaboration, and community engagement should be employed to build competence in applying SDG principles in clinical and public health contexts.

Addressing these gaps through structured curriculum reform and innovative teaching methods may be essential to better prepare future physicians to contribute effectively to achieving the SDGs, especially Goal 3: Good Health and Wellbeing. Further research should evaluate the long-term impact of such interventions and explore their effectiveness across diverse educational settings, including Saudi Arabia's national medical education framework.

## Data Availability

The raw data supporting the conclusions of this article will be made available by the authors, without undue reservation.
